# Quorum-Sensing Inhibition by Gram-Positive Bacteria

**DOI:** 10.3390/microorganisms10020350

**Published:** 2022-02-03

**Authors:** Evgeniya V. Prazdnova, Andrey V. Gorovtsov, Nikita G. Vasilchenko, Maxim P. Kulikov, Varvara N. Statsenko, Anna A. Bogdanova, Alexandr G. Refeld, Yaroslav A. Brislavskiy, Vladimir A. Chistyakov, Michael L. Chikindas

**Affiliations:** 1 Academy of Biology and Biotechnology, Southern Federal University, 344090 Rostov-on-Don, Russia; avgorovcov@sfedu.ru (A.V.G.); nvasilchenko@sfedu.ru (N.G.V.); makkulikov@sfedu.ru (M.P.K.); varstacenko@sfedu.ru (V.N.S.); brislavsky@sfedu.ru (Y.A.B.); vladimirchi@sfedu.ru (V.A.C.); 2 Evolutionary Biomedicine Laboratory, SCAMT Institute, ITMO University, 125993 Saint Petersburg, Russia; aaaabogdanova@gmail.com; 3 Cell Biophysics Laboratory, SCAMT Institute, ITMO University, 125993 Saint Petersburg, Russia; agrefeld@mail.ru; 4 Center for Agrobiotechnology, Don State Technical University, 344002 Rostov-on-Don, Russia; tchikind@sebs.rutgers.edu; 5 Health Promoting Naturals Laboratory, School of Environmental and Biological Sciences, Rutgers State University, New Brunswick, NJ 08854, USA; 6 Department of General Hygiene, I.M. Sechenov First Moscow State Medical University, 119991 Moscow, Russia

**Keywords:** quorum-sensing, quorum-quenching, gram-positive bacteria

## Abstract

The modern paradigm assumes that interspecies communication of microorganisms occurs through precise regulatory mechanisms. In particular, antagonism between bacteria or bacteria and fungi can be achieved by direct destruction of the targeted cells through the regulated production of antimicrobial metabolites or by controlling their adaptive mechanisms, such as the formation of biofilms. The quorum-quenching phenomenon provides such a countermeasure strategy. This review discusses quorum-sensing suppression by Gram-positive microorganisms, the underlying mechanisms of this process, and its molecular intermediates. The main focus will be on Gram-positive bacteria that have practical applications, such as starter cultures for food fermentation, probiotics, and other microorganisms of biotechnological importance. The possible evolutionary role of quorum-quenching mechanisms during the development of interspecies interactions of bacteria is also considered. In addition, the review provides possible practical applications for these mechanisms, such as the control of pathogens, improving the efficiency of probiotics, and plant protection.

## 1. Introduction: Quorum-Sensing and Quorum-Quenching as Mechanisms of Interspecies Interactions

Quorum-sensing (QS) is an intraspecies interaction mechanism characteristic of bacteria and fungi [1]. Quorum-quenching (QQ), or interruption of QS signaling, is a mechanism of interspecies and even cross-kingdom interactions. Bacteria use it as a mechanism of antagonism, while in eukaryotes it is used for protection against pathogens [2]. In a broad sense, QQ refers to any disruption of intercellular communication.

The interruption of a QS signal can occur in several ways: (1) stopping the synthesis of signaling molecules; (2) inactivation or enzymatic destruction of signaling molecules, preventing accumulation to a threshold value; (3) interference with the binding of signal receptors in a bacterial cell or competition with signal molecules—receptor analogs; and (4) blocking target genes that should have been triggered by the QS signal [3,4].

Disruption of the signaling molecules’ synthesis can be achieved by inhibiting the enzymes involved in this process, such as the acyl chain (acyl carrier protein, ACP) and S-adenosylmethionine synthase [4], or, for example, by blocking the Lux system, which serves as a fundamental model for Gram-negative proteobacterial QS systems [5].

Disruption of the signaling molecules’ interaction with receptors can be caused by agonists (analogs of signaling molecules competing with them for receptors) and antagonists (receptor blockers). This mechanism is characteristic of cross-quenching of autoinducers (signaling molecules that are produced as a reaction to changes in population density of bacteria) when each autoinducing peptide (AIP) activates its related receptor but inhibits the activation of all others by competitive binding to foreign receptors. This mechanism is typical, for example, for intraspecific competition between different groups of *Staphylococcus aureus* [4].

The destruction of QS signaling molecules is an enzymatic process. The most well-studied mechanism is the degradation of the autoinducer acyl-homoserine lactone (AHL). This process involves AHL acylases and AHL lactonases, which cut the amide linkage of AHLs and destroy the lactone ring, respectively [5], as well as AHL -oxidoreductase, and AHL-oxidase, which oxidize signaling molecules [6]. The best-studied mechanism is the degradation of AHL by lactonases which are produced by many types of microorganisms. AHL lactonases are grouped into two clusters: the AiiA cluster (produced by *Bacillus*) and AttM (produced by Gram-negative bacteria) [4,5].

From an evolutionary point of view, there are two reasons why bacteria would develop QQ mechanisms. First, it is the mechanism of antagonism with competitors; secondly, it is the utilization of QS signals by bacteria, both to reuse resources and to regulate and “fine-tune” the processes regulated by QS signaling [4].

In addition, enzymes active against QS signals, such as AHL lactonases, have other properties that are beneficial for bacteria, namely, the destruction of toxins. In particular, *Bacillus* lactonases can inactivate mycotoxins such as aflatoxin B1 [7] and zearalenone. Thus, in some cases, QQ activity may be a product of parallel evolutionary processes.

Strategies for the practical use of compounds and strains capable of QQ exist in medicine, animal husbandry, and crop production. Since the manifestation of pathogenicity factors [8], antibiotic resistance [9], and other essential properties of bacteria depend on the work of QS systems, making them the target of therapy seems to be a promising approach.

This work aims to systematize the data on the mechanisms by which Gram-positive bacteria interrupt the QS signals of other bacteria. Among the Gram-positive groups, many probiotic microorganisms enter into a mutually beneficial symbiotic relationship with the host. Studying the mechanisms of their interaction with other representatives of the microbiota may shed light on some aspects of their probiotic activity. The antagonism between probiotic strains and pathogenic microorganisms may be due, in part, to the QQ effect.

## 2. Role of QS in Pathogenicity and Antimicrobial Resistance

The discovery of cell-to-cell communication between bacteria has led to the realization that bacteria can coordinate crucial metabolic activities. For example, in many pathogens, the QS process reduces the host immune response by delaying the production of virulence factors in pathogenic bacteria until the population density becomes sufficient to overcome the host’s defense mechanisms. It is now known that a significant portion of the bacterial genome (4–10%) and proteome (≥20%) is influenced by QS signaling processes. This means that QS is a mechanism used by pathogenic bacteria not only for controlling the production of virulence factors but also for adapting to the metabolic needs for life in the community [8].

### 2.1. QS of Gram-Negative Bacteria

The most studied quorum sensing system among Gram-negative bacteria is the homologous LuxR-LuxI system and related signaling molecules: N-acyl homoserine lactones (AHL). It is similar to the system first studied in the marine bacterium *Vibrio fischeri*. The vast majority of Gram-negative quorum-sensing systems that have been studied so far use N-AHL as signaling molecules. At sufficiently high concentrations, these molecules can bind to a transcription activator or R-protein and activate them, which, in turn, induces the expression of target genes [8]. It is becoming apparent that, in addition to AHL, alternative signaling molecules exist in Gram-negative bacteria. For example, the plant pathogen *Ralstonia solanacearum* produces 3-hydroxypalmitic acid methyl ester as a signaling molecule, which, together with AHL, is used to regulate virulence [10]. *Xanthomonas campestris*, the causative agent of black rot in cabbage, produces a diffusable extracellular factor that has not yet been chemically characterized but is not an AHL [11]. In *Pseudomonas aeruginosa*, a third autoinducer was identified, designated PQS (*Pseudomonas* Quinolone Signal), which differs from two other AHLs produced by this organism [12]. Butyrolactones were isolated from culture supernatants of *Pseudomonas aureofaciens*, and a new family of signaling compounds identified as diketopiperazines (DKP) was found in cell-free supernatants of *P. aeruginosa*, *P. fluorescens*, *P. alcaligenes*, *Enterobacter agglomerans*, and *Citrobacter* [13].

#### 2.1.1. QS in *P. aeruginosa*

Among pathogenic bacteria, *P. aeruginosa* is perhaps the best characterized in terms of its regulated virulence factors and the role that QS plays in pathogenicity. Classified as an opportunistic pathogen, *P. aeruginosa* infections are most common in people with weakened immune systems, such as those with cancer or AIDS, or those with impaired normal barriers caused by burns, persistent medical devices, or long-term use of broad-spectrum antibiotics. *P. aeruginosa* possesses a wide range of both cell-associated and extracellular virulence factors. The expression of many extracellular factors is not constitutive but depends on cell density, with the maximum production of proteolytic enzymes occurring during the late logarithmic and early stationary growth phases. It has been shown that two interconnected QS sensory systems are involved in virulence, biofilm development, and many other processes in *P. aeruginosa*. The first system (Las) consists of a *lasI*-encoded acyl-HSL synthase and a transcriptional activator encoded by *lasR*. The second system (Rhl) consists of an acyl-HSL synthase encoded by *rhlI* and a transcriptional activator encoded by *rhlR* [14]. Recently, a third LuxR-type protein has been identified called QscR (quorum sensing control repressor). Analysis of the *Pseudomonas* genome revealed additional genes encoding LasR and RhlR homologs but did not find genes encoding LasI and RhlI homologs. The predicted ORF is 714 nucleotides long and encodes a polypeptide of 27,236 Da. The QscR protein contains two domains characteristic of LuxR-type regulators: an acyl-HSL-binding domain and a DNA-binding domain. It was found that QscR inhibits the transcription of three QS-controlled gene clusters, *phz* (phenazine), *hcn* (hydrogen cyanide), and *qsc105*. This suppression appears to be effective in the logarithmic growth phase [15].

**Figure 1:**
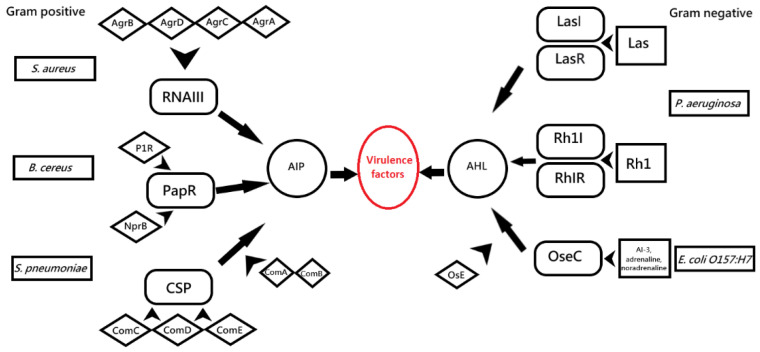
Regulation of QS systems.

QS in *P. aeruginosa* regulates the expression of several virulence factors, and this regulation plays a vital role in pathogenicity (Figure 1). This assumption has been confirmed using several different animal models. In a pneumonia model in newborn mice, the *lasR*-deficient *P. aeruginosa* strain had a significantly lower virulence than the parental one. Another study used three different infection models, *Caenorhabditis elegans* (nematode), *Arabidopsis thaliana* (plant), and *lasR*-deficient mutant mice obtained by random mutagenesis and showed significantly reduced virulence in all three models. These studies prove that the mentioned genes are associated with QS and contribute to the virulence of *P. aeruginosa* in different kingdoms [8,16].

#### 2.1.2. QS in Enterohemorrhagic *Escherichia coli* O157:H7

Infection with enterohemorrhagic *E. coli* O157:H7 (EHEC) can lead to severe gastroenteritis and other extraintestinal manifestations, including fever, meningitis, and sepsis. EHEC also expresses Shiga toxin (Stx) in the gut, whose receptors are also located in the kidney and central nervous system. Stx is a potent inhibitor of protein synthesis and can be systemically absorbed, resulting in hemolytic uremic syndrome (HUS), seizures, cerebral edema, and coma. 

There are three signals in EHEC that may activate the transcription of virulence genes: a bacterial aromatic autoinducer (AI-3) produced by the normal microbiota of the gastrointestinal tract and two hormones (adrenaline/norepinephrine) produced by the host. These signaling molecules can activate a sensor associated with QseC, a membrane-localized receptor (sensor kinase, leading to the transcription of virulence genes. QseC also activates the expression of a second gene, *qseE*, which helps fine-tune the signaling cascade (Figure 1). All these transcriptional events lead to the formation of lesions in the intestine and the production of Stx [17].

### 2.2. QS in Gram-Positive Bacteria

The vast majority of QS-related studies are focused on Gram-negative species of bacteria. However, many Gram-positive pathogens pose a serious health threat and their virulence has been shown to be QS-mediated. For instance, QS is involved in the pathogenesis mechanisms of *Bacillus cereus*, *Streptococcus pneumoniae*, and *S. aureus* [8].

The nature of the signaling molecules used in Gram-positive QS-systems is different from that of Gram-negative organisms. QS systems in Gram-positive bacteria usually use small signal peptides that have undergone post-translational processing. These peptide signals interact with the sensory element of a two-component histidine kinase signaling system. It was found that Gram-positive bacteria have two families of transcription factors, RNPP and Rgg, which have a binding domain. RNPP is a protein family which stands for Rap, NprR, PlcR, and PrgX. This family includes all Gram-positive QS systems which bind directly to their signaling peptide in the recipient cell. RNPP is a critical factor in regulating several processes such as sporulation, conjugation, biofilm formation, and pathogenic responses.

The Rgg (regulator gene of glucosyltransferase) transcription factors directly bind pheromones transported to the cytosol (Figure 1). Rgg-like regulators (a family of transcription factors) are found in most gram-positive bacteria. In different species, the Rgg protein can be a regulator of extracellular glucosyltransferase expression (*L. lactis*), control antibiotic expression (*Streptococcus mutans*), or control protein secretion (*S. pyogenes*) [18]. Thus, Rgg can influence the transcription of natural resource use and provide adaptation to environmental conditions. Due to the different organization of Gram-positive QS-systems, alternative QQ mechanisms are utilized to intercept or block signaling in these species [19].

#### 2.2.1. QS in *S. aureus*

*S. aureus* is a normal constituent of the microbiota of several ecological niches of the human body, including on the skin and the mucous membranes of the respiratory tract. When epidermal or mucosal barriers are damaged or broken, *S. aureus* can cause a wide range of diseases, from minor skin infections to life-threatening pneumonia, bacteremia, and sepsis. This is particularly prevalent in health care settings, with *S. aureus* being the leading cause of nosocomial infections [7].

The ability of pathogenic *S. aureus* to cause disease depends on the expression of various adhesion molecules, toxins, and compounds that affect the immune system. QS regulates the expression of genes encoding these virulence factors. *S. aureus* uses the canonical Gram-positive two-component QS system encoded by the *agr* locus [19]. The additional gene regulator (*agr*) system consists of RNA, RNA II, and RNA III transcripts. The RNAII operon system consists of four genes: *agrB*, *agrD*, *agrC*, and *agrA*. The signaling cascade begins with the production of a 46 amino acid peptide encoded by the *agrD* gene, which is then modified by the integral membrane protein AgrB. The modified peptide acts as a final autoinducing peptide (AIP). AgrA and AgrC together form a two-component system with an AIP binding domain and act as a histidine kinase. Induction of the two-component system activates the RNAII operon and acts as a transcription factor for the RNAIII transcript itself [20]. RNAIII has a dual function: it activates the production of α-toxin and suppresses the expression of fibronectin-binding proteins A and B, peptide A, coagulase, and other surface proteins. The result of this QS regulatory cascade is the suppression of surface virulence factors (such as peptide A) and an increase in the level of secreted virulence factors (such as alpha toxin) (Figure 1). Another critical component of *S. aureus* virulence is biofilm formation. In *S. aureus*, the *agr* system regulates biofilm formation. One interpretation of this finding is that the formation of a biofilm gives *S. aureus* time to grow to a certain density, at which point it is optimally ready to secrete virulence factors. To facilitate cell proliferation, *S. aureus* stops biofilm formation and reduces surface proteins and adhesion. This behavior is similar to a strategy used by *V. cholerae* [21]. Concerning weakening the expression of virulence genes, known strategies can be grouped into different categories: (1) inhibition of the catalytic functions of AgrB and SpsB, (2) competitive inhibition of AgrC using natural QSI agents or synthesized analogs of AIP, (3) blocking the activity of histidine kinase, (4) inhibition of AgrA P2/P3 interactions; and (5) inhibition of RNAIII transcription [22].

#### 2.2.2. QS in *Bacillus cereus*

Some strains of *B. cereus* are pathogenic bacteria that can colonize the intestines and produce several virulence factors, including hemolysin, causing abdominal pain and diarrhea. The transcription factor PlcR, along with AIP, regulates the genes for the secretion of the virulence factor in *B. cereus*. The initiation of the QS signaling pathway begins with the expression of the *papR* gene. The translation product of the *papR* gene is a 48 amino acid peptide (PapR), which is then transported outside the cell by a membrane carrier protein. The PapR peptide is then cleaved by the catalytic activity of protein B (product of the *nprB* gene) into four small peptides capable of activating the *plcR* gene [23]. The last AIP enters the cell again through the membrane channel protein, binds to the PlcR protein, and stimulates the expression of several genes for the secretion of virulence factors. The *plcR* gene product also acts as an inhibitor of biofilm formation, but the detailed mechanism is still unclear [24]. However, it was found that inactivation of the *plcR* gene reduces the secretion of virulence factors but cannot eliminate virulence factor secretion since several additional systems are involved in QS and the regulation of virulence factors (Figure 1). These additional sensory inputs include sporulation via SpOA P, feeding via CodY, motility via FlhA, and other two-component systems [19].

#### 2.2.3. QS in *Streptococcus pneumoniae*

*S. pneumoniae* is a pathogenic bacterium that causes pneumonia, otitis media, and meningitis in humans. Like other Gram-positive bacteria, *S. pneumoniae* also uses a 17-residue signal peptide called competence stimulating peptide (CSP). In general, *S. pneumoniae* exhibits two gene regulation processes/pathways: early gene expression (regulated by ComE) and late gene expression (regulated by ComX). There are two sets of genes that are expressed over time, and the expression of both sets of genes leads to the development of competence. The ComCDE operon plays a decisive role in developing competence and biofilm formation in *S. pneumoniae* [25]. The *comC* gene encodes immature CSP molecules in the cell, while the products of the other two genes, ComD and ComE, act as transmembrane receptors for CSP molecules and a response regulator system, respectively. The transformation of immature CSP molecules into active ones is carried out using ComA and ComB. The transmembrane channel protein, an ABC transporter, actively transports CSP molecules outside the cell, acting as an inducer of the QS pathway. The binding of CSP to its ComD receptor transfers the phosphate group to ComE. Activated ComE acts as a transcription factor for several genes such as *comAB*, *comC*, and *comDE* (Figure 1). It is also reported that ComE is a transcription factor for the *comX* gene. This gene produces sigma factor and ComW, which are necessary for the development of pathogenicity [26].

Although few examples of QQ against streptococci have been found [27], the mechanisms of their QS systems are typical for many pathogens, and it is hoped that the search for QS inhibitors against streptococci will become a promising direction of research in the future.

### 2.3. Regulation of Microbial Resistance with QS

#### 2.3.1. Efflux Pumps

Efflux pumps play an important role in the formation of multidrug resistance in bacteria [28]. The regulatory effect of the QS system on the expression of multidrug-resistant pumps is that the expression of the pumps can be regulated, while the QS system itself also depends on the expression level of the pumps. Thus, it was found that in *Bacteroides fragilis*, when cultivated in the presence of self-induced molecules C6-HSL and C8-HSL, the expression of the efflux pump operon’s *bmeB* increased, and resistance to antibiotics was achieved [29]. It was also found that an autoinducer can activate the multidrug resistance pump MexAB-OprM, developing multidrug resistance in bacteria. As mentioned above, the QS system itself also depends on the level of efflux pump expression. However, some researchers have found that overexpression of the MexCD-OprJ pump turns off the *P. aeruginosa* QS response [30]. When some efflux pumps (such as the RND-resistance-nodulation-division superfamily) displace the antibiotic from the cell to form drug resistance, self-induced molecules of the QS system can also be displaced from the cell, increasing the concentration of self-induced extracellular molecules, which can exacerbate bacterial infections [28]. This finding suggests that high expression of the efflux pump can further activate the QS system, promote the regulation of the synthesis of the toxin factor of infection and the expression of the efflux pump by the QS system, and increase the infectivity and invasiveness of pathogenic bacteria.

#### 2.3.2. Biofilms

Recent studies have shown that most human bacterial infections are associated with biofilms, the formation of which is one of the important reasons why clinical bacterial infections are difficult to treat [31]. It was found that bacterial biofilms can lead to bacterial drug resistance due to penetration restriction, nutritional restriction, and phenotypic mechanisms of drug resistance. The molecular barrier and the biofilm (mostly negatively charged) formed by polysaccharides can prevent or slow down the penetration of certain antibiotics, which is the primary mechanism for limiting permeability. Biofilm development provides resistance to desiccation, oxidative stress, and the action of proteases and antimicrobial agents [19]. In Gram-positive bacteria, the QS system regulates biofilm formation using oligopeptides as signaling molecules that can be recognized by a two-component sensory protein after modification and affect the expression of the target gene through phosphorylation and dephosphorylation of the protein [24].

In general, the regulation of QS systems can be summarized in the following scheme (Figure 1).

## 3. Mechanisms and Metabolites That Are Responsible for QQ in Different Groups

### 3.1. Firmicutes

#### 3.1.1. *Bacillus*

The most common bacterial strains, including soil bacilli capable of inhibiting biofilm formation, can be found in the rhizosphere of plants, various bodies of water, and other microbial communities. For example, various authors have shown that a high abundance of AHL-degrading bacteria can be found in the rhizosphere of potatoes, mainly belonging to the genera *Agrobacterium*, *Bacillus*, *Pseudomonas*, *Delftia*, *Ochrobactrum*, and *Rhodococcus* [32,33].

The majority of studies on quorum-quenching have focused on the enzymatic degradation of AHL. Studies in this area have identified three main categories of AHL degradation enzymes that correlate with their enzymatic mechanisms [34]. The enzyme categories and their molecular mechanisms are presented in Table 1.

The enzymes and enzyme complexes listed above can degrade AHL and, as a result, prevent pathogenic bacteria from producing virulence factors and forming biofilms, thereby reducing their virulence.

**Figure 2:**
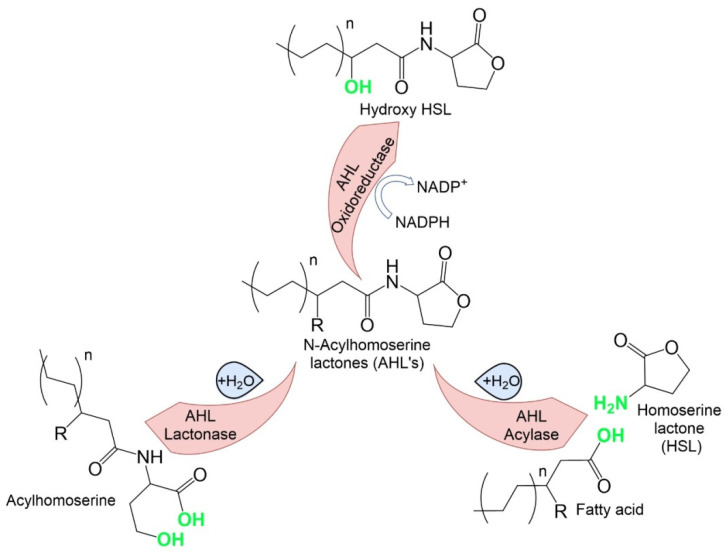
AHL enzymes and their molecular mechanism.

The differences between the mechanisms of the enzymes that degrade AHL’s can be seen more clearly in the figure below (Figure 2).

The first bacteria capable of degrading foreign QS signals were isolated from soil and subsequently identified as members of the genus *Bacillus* [35]. Spore-forming bacteria of the genus *Bacillus* have long been known for their high antagonistic potential against a wide range of microorganisms. These bacteria are often referred to as biological control agents and plant growth-promoting bacteria [36,37].

Members of the genus *Bacillus* have many desirable properties as biocontrol agents, such as ease of cultivation, long-term storage as preparations (due to the ability for spore formation), wide temperature tolerance, etc.

The ability to control the growth of other microorganisms by bacteria of this genus is due to both the production of a wide range of antibacterial and antifungal molecules [38] and the ability to suppress communication inside biofilms and fungal cell groups by quorum-quenching molecules, as will be shown below. At present, many authors have shown that bacilli actively inhibit foreign QS signals and can be isolated from various microbial communities.

For example, Dong et al. [35] showed that *B. thuringiensis*, *B. cereus*, and *B. mycoides* strains isolated from soil and plant samples exhibited a high potential to inactivate AHL. It is known that the listed bacterial species belong to the *B. cereus* group. Because of this, the subsequent work of these authors focused on the bioinformatic search for AHL-lactonase homologs in bacteria also belonging to this group. The authors’ analysis revealed an AHL-lactonase homolog in *B. anthracis* (*gnl/TIGR_1392/banth_2063*), in which 89.1% of the nucleotides are identical to those in the aiiACOT1 coding sequence, suggesting that *B. anthracis* may contain a similar AHL-inactivating enzyme. However, the role of AHL lactonase in *B. anthracis* is not completely clear.

Anandan and Vittal (2019) [39] isolated the endophytic *Bacillus thuringiensis* strain KMCL07 with high lactonase activity against *Pseudomonas aeruginosa* lactones from plants. Further study of the isolated strain concluded that its high QQ activity was due to the production of lactonase AiiA, which belongs to the metal-β-lactamase superfamily [39].

QQ in the rhizosphere likely plays a major role in PGPR (plant growth-promoting rhizobacteria) and plant interactions and likely has a significant impact on plant health through the suppression of pathogenic microbiota in the plant rhizosphere.

Although, for the most part, AHLs have more of an indirect negative effect (by increasing pathogen biomass and, consequently, causing more severe forms of the disease). However, various studies have shown that the presence of AHLs in the rhizosphere directly induces various functional and some beneficial responses in various plants [40,41,42]. This shows the importance of AHL from beneficial bacteria in the rhizosphere for better growth and development of plants, demonstrating the role of QS signals as cross-kingdom messengers. Probably, to prevent the reproduction of a bacterial group that is antagonistic to its species, but at the same time not to suppress harmless ones, bacteria capable of quenching foreign QS signals must have recognition systems that trigger a response to external threats.

For example, the authors of one study demonstrated that *B. subtilis* induces the expression of the *ytn*P gene only in the presence of streptomycin, an antimicrobial agent produced by the Gram-positive bacterium *Streptomyces griseus*, which would threaten the survival of a *Bacillus* population. The *ytn*P gene encodes a lactonase homologous protein that can inhibit the signaling pathway required for streptomycin production and aerial mycelial development in *S. griseus* [43].

This behavior serves as a protective strategy against antagonistic bacteria because it allows *B. subtilis* to inhibit the QS system of harmful microbial communities selectively. Selective inhibition of QS would not occur if *B. subtilis* triggered *ytnP* expression in response to the presence of lactone molecules. To ensure that the QQ activity by YtnP acts only in the presence of antibacterial molecules or other stressors that disrupt *B. subtilis* cell physiology, YtnP is expressed as a cytoplasmic enzyme, just like other quorum-quenching enzymes [43]. Consequently, the release of YtnP into the extracellular space co-occurs with cell lysis, which is a peculiar mechanism for regulating the activity of a QQ system in the rhizospheric coexistence of soil bacilli with other community members.

In addition to soil and plant-associated bacilli, the production of AHL-degrading enzymes was also found in strains isolated from marine water communities. McBride and Strickland [44], for example, showed that the most active of the sediment strains they isolated was *Bacillus pumilus* S8-07. This bacterial strain reduced the accumulation of N-acyl homoserine lactones (AHLs) and showed significant inhibition of the production of LasA protease, LasB elastase, caseinase, pyocyanin, pyoverdine, and biofilm formation in *P. aeruginosa* PAO1, and showed a significant decrease in prodigiosin synthesis, secreted caseinase, hemolytic activity, and biofilm formation in *Serratia marcescens* [45]. It is also worth noting that the authors of this work were the first to demonstrate that QQ-activity can be caused not by the action of lactonase, as has often been demonstrated in other works, but by the action of an enzyme with acylase activity in bacilli.

In another study, the authors isolated a quorum-quenching *Bacillus* sp. Strain, designated as QSI-1, from the intestines of healthy fish (*Carassius gibelio*). QSI-1 was found to inhibit AHL-dependent violacein production in *Chromobacterium violaceum*, reduce the production of virulence factors such as proteases, hemolysins, and inhibit biofilm formation in *Aeromonas hydrophila* YJ-1 [46]. The data presented by these authors also allows considering *Bacillus* representatives as probiotic strains for aquaculture.

Although most studies are focused on quenching QS signals of a lactone nature, data on the ability of *Bacillus* bacteria to interact with other signaling molecules, including mediators of QS in Gram-positive bacteria, have recently been published. For example, it was shown that *B. subtilis* could inhibit QS in *S. aureus* due to the competitive interaction of the *Bacillus* synthesized lipopeptide fengicin with AgrC, a receptor for staphylococcal AIP. At the same time, comparative studies in human populations indicate that the effect is not limited to the intestinal microbiome but is systemic: in a rural Thai population, consuming probiotic *B. subtilis* led to the eradication of *S. aureus* in the nasopharynx. This may be related to the anti-biofilm effect achieved by the synthesis of molecules with QQ-activity [47]. *Agr*-like QS-systems also exist in other pathogenic bacteria, particularly in *Clostridium perfringens*, which causes necrotizing enteritis in chickens [27]. Recent work has shown that this is also characteristic of the important human pathogen *Clostridioides difficile* [48]. At the same time, no enzymatic systems that suppress QS in this pathogen have yet been identified. Still, the similarity of the QS system with that in *S. aureus* allows us to hope for the successful use of probiotic *Bacillus* strains. The ability of subtilisin A to block QS in the opportunistic actinobacterium *Gardnerella vaginalis* through interaction with the autoinducer AI-2 has also been shown [49].

In summary, it is worth emphasizing that bacteria of the genus *Bacillus*, as this chapter shows, are the most promising targets for the study of QQ-systems since they have ubiquitous occurrence and various mechanisms for the enzymatic degradation of QS-signaling Gram-negative bacteria, namely production of AHL-lactonases, and as shown relatively recently, AHL-acylases. The search for strains capable of effectively counteracting the QS systems of Gram-positive bacteria represents a new and very pressing challenge that has yet to be addressed.

#### 3.1.2. Lactic Acid Bacteria as Quorum-Quenching Agents

Since ancient times, lactic acid bacteria (LAB) have played an essential role in human life. Many types of lactic acid bacteria are used to create traditional food in different countries. Lactobacilli are an integral part of the normal intestinal microbiota of humans and other animals. Also, lactobacilli, especially *Lactobacillus acidophilus*, *Lactobacillus delbrueckii* subsp. *lactis* (formerly *Lactobacillus lactis*), *Lactiplantibacillus plantarum* (formerly *Lactobacillus plantarum*), are some of the most popular probiotics.

Mainly, when talking about the benefits of lactic acid bacteria, it comes to the fact that they produce lactic acid and other substances (bacteriocins, hydrogen peroxide, diacyls, and others) that inhibit the growth of other bacteria. Lactic acid lowers the pH of the medium, which is detrimental to the development of putrefactive bacteria. Lactic acid bacteria also often play an immunomodulatory role as symbionts of the gastrointestinal tract. For example, lactic acid causes macrophage polarization in the anti-inflammatory M2 phenotype [50].

Lactobacilli have a wide range of properties, in particular, antagonism to other microorganisms. Nonetheless, they are less commonly regarded as QS regulators of pathogens than other Gram-positive bacteria such as bacilli. However, there is evidence showing the potential activity of lactobacilli as QQ agents. As we will discuss further, lactic acid bacteria can potentially affect QS, mediated by acylated homoserine lactones (AHL, HSL) and furanosyl borate diester (also known as autoinducer 2 or AI-2)

A recent review by Gunaratnam S. et al. (2021) [48] describes how some probiotic strains of LAB exhibit inhibitory activity against the QS system and its processes in Gram-negative bacteria. However, it is also possible to add information to the discussion. Today, there is growing evidence concerning the QQ activity of LAB, though it’s still unclear which molecules play a key role.

In 2018, J. Kim et al. [51] showed that the probiotic *L. acidophilus* 30SC can disrupt biofilm formation in an *E. coli* O157:H7 strain. In the presence of the cell extract of *L. acidophilus* 30SC, the level of biofilm formation was significantly lower than that of wild-type *E. coli* and approached the level of an *E. coli* O157:H7 *luxS* isogenic mutant, which is unable to produce AI-2.

It was found that *L. acidophilus* 30CS can also significantly inhibit the AI-2 activity of *E. coli* O157:H7. It was discovered using a bioluminescence test based on *Vibrio harveyi* strain BB170 acting as a positive control of the AI-2 sensor signal [51,52].

In an earlier work by J. Kim et al. (2012) [53], it was shown that some cell extracts of *Bifidobacterium* spp. have similar activity, although, strictly speaking, bifidobacteria are not LAB. They can also suppress biofilm formation and AI-2 activity in *E. coli* O157:H7. However, comparing these results with the similar work discussed above makes it clear that *L. acidophilus* has more significant inhibitory activity and can claim to be a more promising QQ agent.

Interestingly, there is evidence of the ability of various *Lactobacillus* species to inhibit the activity of various acyl-homoserine lactones. For example, in a study by Kampouris et al. (2018) [54] focused on solving the problem of overgrowth on filtration membranes, it was shown that lactic acid bacteria can inhibit N-Hexanoyl-L-homoserine lactone (6-HSL). These lactic acid bacteria were isolated from activated sludge, identified as *L. plantarum*, and encapsulated in alginate beads. The maximum results were observed in *L. plantarum* strain SBR04MA. Within 9 h, this strain degraded all 6-HSL. It also successfully suppressed the biofilm formation of other species from activated sludge and, as a result, suppressed the synthesis of various exogenous polysaccharides that clog the pores of the filter membrane.

It has been shown that *L. plantarum* CY-1 can act as a QQ agent against *Aeromonas sobria*, which is a common pathogen in aquaculture [55]. *L. plantarum* CY-1 is able to synthesize ASH of various lengths: C4, C6, C8 ++, C10 ++, C12 [56]. However, the ratio of “non-classical” carbon lengths to the more common C4- and C6-HSL is small.

In previous work by Ly et al. (2021), it was shown that different strains of *L. plantarum* degrade HSL, but the type of HSL was not specified. To determine whether the activity was inhibited, a test with *Chromobacterium violaceum* was used, which releases the violet pigment violacein in response to the presence of HSL. However, at the moment, it is known that entirely different types of HSL can act as inducers for the synthesis of violacein [57]. Nevertheless, a strain was isolated that is capable of degradation of HSL by almost 100%. In this regard, it was shown that the strain inhibits the development of biofilms and affects the synthesis of virulence factors.

Cui T. et al. (2020) [58] showed that *Lactobacillus crustorum* ZHG 2-1 (re-classified as *Companilactobacillus crustorum*) isolated from pickled cucumbers has a similar effect on *P. aeruginosa*. Cell extracts of this strain are capable to destroy two types of HSL: C4 and 3-oxo-C12 dose-dependently (1 mg/mL, 2 mg/mL and 3 mg/mL caused degradation of C4-HSL 37.13%, 55.36% and 76.28%, respectively and degradation of 3-oxo-C12-HSL 43.25%, 63.71% and 87.62%, respectively). Inhibition of biofilm formation, reduction of swarming and swimming motilities, and inhibition of virulence factors (chitinase and protease) were also observed without affecting bacterial growth.

There is another example of the influence of lactic acid bacteria metabolites on QS in *P. aeruginosa*. Rana et al. (2020) [59] investigated the effect of lactic acid bacteria’s acidic and neutral cell-free supernatants on QS and QS-associated processes. The effect differed both between the types of supernatants and between the methods of their use. For example, the most exciting results were shown by the acidic supernatants of *Lactococcus lactis* NCDC 309, which were added to the medium inoculated with pseudomonads only. In this case, effective inhibition of biofilm, AHL, elastase, and *lasI* and *rhiI* expression was observed.

Interestingly, neutral supernatants of all studied strains have a controversial effect. On the one hand, these supernatants effectively degraded AHL (5-10 times compared to the control), and on the other hand, they stimulated or did not affect elastase activity. What is especially interesting, is that the neutral supernatants of *Lactobacillus fermentum* MTCC 5898 (reclassified as *Limosilactobacillus fermentum*), *Lactobacillus rhamnosus* MTCC 5897 (reclassified as *Lacticaseibacillus rhamnosus*), and *L. lactis* NCDC 309 in different cases showed an increase in the expression of *las*I and *rhi*I in comparison with the control.

To date, no data revealing the mechanisms of QQ by LAB in detail is available. It could be assumed that lactonases are responsible for the destruction of AHL in LAB. According to Rana et al. (2020) [59], the acidic supernatant with pH 4.0 had a more pronounced AHL-degrading activity. However, there is data showing AHL lactonase has the optimal pH range of 6-8, which corresponds to a more neutral environment [60,61]. Interestingly, the molecules themselves are more stable in an acidic environment, and they undergo non-enzymatic lactonolysis at pH between 5–8 [62]. It should be noted that there is no convincing evidence that lactic acid bacteria, particularly lactobacilli, are capable of producing AHL lactonases. This suggests that, most likely, other molecules that have not yet been identified are involved in the degradation of AHL in LAB.

In addition to the ability of lactobacilli to suppress QS-signaling in Gram-negative bacteria by interacting with various types of AHL, there are many studies devoted to their interaction with QS-signals from Gram-positive bacteria. The most significant number of works is devoted to the suppression of QS in *S. aureus*. For example, culture supernatants of *Lactobacillus reuteri* RC-14 (reclassified as *Limosilactobacillus reuteri*) suppressed the expression of *agr* genes, and cyclic dipeptides cyclo (L-Phe-L-Pro) and cyclo (L-Tyr-L-Pro) played a crucial role in this inhibition [63]. Yan et al. (2019) [64] showed that biosurfactants produced by *L. plantarum* and *P. acidilactici* reduce the expression of the *cidA*, *icaA*, *dltB*, *agrA*, sortase A, and *sarA* genes associated with biofilm formation in *S. aureus*, as well as reducing the expression of AI -2 in a dose-dependent manner.

Among other Gram-positive pathogenic bacteria, QS in *Listeria monocytogenes* was inhibited by the metabolites of *L. plantarum* M.2 and *Lactobacillus curvatus* B.67 (reclassified as *Latilactobacillus curvatus*) due to inhibition of *agr* genes [65]. A similar mechanism has been noted for *C. difficile*, which has been shown to inhibit AI-2 and the QS (*luxS*) system upon adding heat-treated supernatant *L. fermentum* Lim2 [66]. These data may indicate the low molecular nature of the metabolites involved in QQ of Gram-positive bacteria in lactobacilli. The information about LAB strains capable of QQ is summarized in Table 2.

The question remains: why do different strains affect certain forms of QS? As can be seen from the discussion and the summary table above, some strains can inhibit different types of AHL, others—AI-2 signaling. In the authors’ opinion, this issue is not fully resolved and is not yet clear, because usually only one of the inhibitory pathways is studied: either against AHL or AI-2, as well as against the processes associated with QS in general (biofilm formation, the activity of pathogenic factors, etc.). In addition, the study of AI-2 inhibition comes down to using a biosensor to understand whether there is inhibition or not and how strong it is. However, knowledge about the nature of this inhibition remains exceptionally vague, especially in lactic acid bacteria.

### 3.2. Actinobacteria

Actinobacteria are a widespread type of microorganisms showing a broad phenotypic diversity, making them indispensable for the biotechnology industry. They play an essential role in the soil ecosystem due to their ability to decompose organic matter. In addition, actinobacteria are widely distributed in the rhizosphere and produce a wide range of biologically active metabolites that affect plant development [68]. Their ability to synthesize a wide range of secondary metabolites such as antibiotics and fungicides [69,70] and their ubiquity make them ideal biological control agents. Actinobacteria are often considered to be regulators of QS.

Actinobacteria are a promising biocontrol agent for reducing soft rot and blackleg caused by *Pectobacterium carotovorum* subsp. *carotovorum* [71]. In a study of 40 marine actinobacteria isolates, 10 had QQ activity. These isolates were able to inhibit biofilm formation in *P. aeruginosa* ATCC 27853, *S. typhimurium*, *V. cholerae*, *B. cereus* ATCC 14579, *S. aureus* ATCC 29213, and *E. faecalis* ATCC 33186 [72].

In a different study 8 *Actinomycetes* isolates had the ability to inhibit biofilm formation in *B. cereus*, *B. subtilis*, and *S. putrefaciens*, presumably due to their ability as anti-QS agents. QQ activity was determined by treating *C. violaceum* with isolate extracts and measuring the degree of inhibition of violacein production [73]. Also, screening of soil and endophytic actinomycetes for QQ activity was attempted, and it was shown that 51.5% of the strains studied possessed this activity, with 36.9% more such activity occurring among endophytic strains [74].

Several genera of this phylum showed the ability to inhibit QS. For example, *Arthrobacter* sp. demonstrated the ability to degrade 3-oxo-C6-HSL and some other N-acyl-homoserine lactones with the help of AhlD lactonase. A co-culture experiment demonstrated the ability to reduce the virulence of the soft rot pathogen *P. carotovorum* N98 [75].

*Microbacterium testaceum* demonstrated the ability to inhibit exoenzyme release by the potato soft rot pathogen *P. carotovorum* subsp. *Carotovorum*, which is induced by AHL-mediated QS. *Microbacterium testaceum* can degrade AHL using AiiM, an AHL lactonase from the Fold family of hydrolases. *M. testaceum* is an endophytic bacterium; given its antipathogenic activity, it can be used as a biological control agent [76].

The ability of *Micromonospora* sp. TAV14 to inhibit *P. aeruginosa* PAO1 swarming by disrupting the rhl-regulated rhamnolipid responsible for swarming has also been shown [77].

It has also been reported that *Streptomyces* sp. can synthesize AHL acylase, which explains their ability to inhibit pathogen virulence factors. The ahlM gene encodes its AHL acylase, showing some similarity to the AHL acylases of strains *Ralstonia* XJ12B (AiiD) and *P. aeruginosa* PAO1 (PvdQ) and the cyclic lipopeptide acylases of strains *Streptomyces* sp. FERM BP-5809 and *Actinoplanes utahensis* [78]. Based on these findings, we can conclude that AhlM may have the ability to degrade not only AHL but also cyclic lipopeptides. In *P. aeruginosa*, elastase, LasA-, and casein protease activities are reduced under the influence of AhlM [79]. In another study, *Streptomyces* sp. NIO 10068 and *Streptomyces* sp. NIO 10058 extracts containing cinnamic acid as the main compound was found to inhibit *P. aeruginosa* ATCC 27853 [80]. As in the case of *Micromonospora sp*, this activity is due to its effect on rhl-regulated rhamnolipid synthesis [77].

*Rhodococcus erythropolis* is of particular interest because all 3 types of AHL degradation enzymes, AHL lactonase, acylase, and oxidoreductase are produced, which is very rare. Genomic studies show that besides *R. erythropolis*, only three other organisms possess AHL-lactonase and AHL-acylase activities together: *Deinococcus radiodurans*, *Hyphomonas neptunium*, and *Photorhabdus luminescens* subsp. *Laumondii* [81]. In addition, the AHL lactonase of *R. erythropolis* belongs to a unique class, unlike the common AiiA-type lactonases. Its lactonase QsdA (Quorum-sensing signal degradation) belongs to phosphotriesterases and is capable of degrading a wide range of AHL [82,83]. A wide range of AHL signaling molecules is degraded by AHL acylase and AHL oxidoreductase for which specific genes have not yet been identified [84]. Another interesting point is that according to C. Barbey [85], QQ activity in *R. erythropolis* is induced by the presence of QS molecules, namely AHL, in the environment. QS activity is regulated by the *qsd* (QS signal degradation) operon, which includes genes encoding lactonase QsdA and fatty acyl-CoA ligase QsdC, which are involved in AHL degradation by affecting the lactone ring and acyl chain fragments. The operon is regulated by the QsdR repressor, a member of the TetR-like receptor family. Using interacting approaches and a transcriptional fusion strategy, they elucidated the mechanism of repressor removal. The homoserine lactone ring binds to the effector domain of QsdR, thereby preventing QsdR from binding to the *qsd* promoter region [86].

The information about *Actinobacteria* strains capable of QQ is summarized in Table 3.

Based on the above, we can say that actinobacteria are promising subjects for the study of QQ. Also, this type has several bacteria shown to be promising agents for controlling Gram-negative plant pathogens whose pathogenicity is due to QS signals.

## 4. The Evolutionary and Ecological Role of QQ

Quorum-quenching, being an important process in the vital activity of microorganisms, has considerable ecological and evolutionary significance. Indeed, if we consider that microorganisms are ubiquitous, and in the course of hundreds of millions of years of evolution, have competed with each other for certain eco-niches, we can expect the influence of QS systems and their suppression on the evolution of some groups of bacteria.

One of the essential aspects of this effect is the reduction of the protection of the population exposed to QQ from bacteriophage infection. It has been proven that QS plays a role in increasing protection against phages. So, on the one hand, given the prevalence of bacteria in the form of a biofilm, it would be logical to assume that bacteriophages have “learned” to effectively infect bacteria in biofilms. Indeed, phages can produce enzymes that destroy the extracellular matrix and, in general, cope well with a biofilm barrier [86].

However, it has also been shown that a biofilm can still prevent or slow down phage infection in the population, hindering the penetration of phages. Microorganisms take protective measures in conditions of high population density and, accordingly, high risk of infection [52]. It is known that the interaction of bacteriophages with bacterial biofilms proceeds along different paths and depends on many factors (phage size, its specificity, the presence of enzymes that destroy the biofilm in the phage, the composition of the biofilm itself, its density) [87].

QQ has an overwhelming effect on the molecular signals responsible for countering phage infection. For example, the QQ mechanism suppresses biofilm formation [5]. Suppression of biofilm formation under the action of QQ enzymes has been actively studied [88]. In this way, some bacteria are capable of weakening the anti-phage defense of their competitors. After eliminating competitors by phages, these bacteria can occupy their niches (the high specificity of bacteriophages allows minimizing the negative consequences for a competitor using the QQ mechanism, with negative consequences for a bacterium with a suppressed QS system and, consequently, weakened protection against phages).

Thus, QQ influences both the ecology of phages and the evolution of host bacteria. It is well known that bacteriophages play a crucial role in horizontal gene transfer between microorganisms [89]. In the context of microbial ecology and the relationship between groups of Gram-positive and Gram-negative bacteria, many examples of QS suppression in Gram-negative bacteria are known [90]. In this case, suppression of biofilm formation in Gram-negative bacteria allows slower-growing Gram-positive bacteria to successfully colonize microniches in environments such as soil and the rhizosphere.

Even though QQ is often used in the competition of microorganisms, this effect also has the role of cleansing the microenvironment of bacterial signaling molecules (the bacterium itself, which produced the QS signal, suppresses, or destroys the signal). It is necessary to reduce the quantity and concentration of information signals around the bacterium—after all, a large number of old signals can interfere with the perception of new, more important ones. Therefore, the ecological effect of QQ as a purification from “superfluous” information around is no less important than the effect of QQ in competition [2].

Considering the evolutionary aspect, we should mention the phylogenetic relationships of various genes (proteins) responsible for QQ and QS. Such relationships were explored in the study of Kalia V.C. et al. [91]. Their study examined the phylogeny of AHL acylases (cleavage of the acyl chain) and AHL lactonases (hydrolysis of the lactone ring). In particular, it was found that AHL-lactonase is present in large quantities among representatives of *Firmicutes* and α-proteobacteria (in general, it provides these enzymes, distributed between groups: *Actinobacteria*, *Acidobacteria*, *Bacteroidetes*, *Chloroflexi*, *Deinococcus-Thermus*, *Firmicutes*, *α-Proteobacteria*, *β-Proteobacteria*, *γ-Proteobacteria*, *δ-Proteobacteria*, *Euryarchaeota*, *Crenarchaeota*, *Sphingo-bacteria*, *Spirochaetales*, *Nitrospirales* and *Planctomycetes*).

It was also shown that the distribution of sequences for AHL-acylase (the frequency of occurrence, for example, in many proteobacteria) was more limited in comparison with the distribution of sequences of AHL-lactonase.

On the other hand, cyanobacteria were found to possess only AHL acylases, while *Acidobacteria*, *Sphingobacteria*, *Spirochaetales*, *Nitrospirales*, and *Planctomycetes* possess only AHL lactonase. In the course of studying the sequences encoding these enzymes, regions were found that are specific for specific taxonomic classes, as well as regions that are universal for different taxonomic classes. In summation, there is an unevenness in the distribution of genes of lactonases and acylases between strains of different taxonomic groups of microorganisms, and in the sequences themselves, there are areas common to large taxa and variables.

As can be seen, the evolution of the strains was accompanied by a parallel evolution of the QQ genes, during which the variable regions of the lactonase and acylase genes changed in the zone of the variable regions [91].

It is also possible to trace the evolutionary relationship between the genes of QS synthases. These genes are similar in phylogenetically close strains of microorganisms. For example, the study by Goh et al. [92] carried out a phylogenetic analysis using the example of *Citrobacter amalonaticus* L8A QS synthases.

The QQ effect is used not only in interactions between microorganisms but also during cross-kingdom interactions: plants can also suppress the QS signals of bacteria. For example, they can effectively suppress information signals from plant pathogens and thus protect themselves from pests (many plant extracts have been tested to suppress QS in bacteria and have shown a QQ effect). However, it cannot be said unequivocally that the suppression of QS bacteria by plants has only a positive effect—in some cases, bacterial QS signals are aimed at triggering the expression of bacterial genes that promote plant growth and protection (for example, the production of antibiotics and antimycotics to protect the plant from the pathogen). In this way, by suppressing QS signals aimed at maintaining a healthy plant life, it is possible for the plant to indirectly cause harm to itself [2].

Another important aspect of the effect of QQ on the metabolism of microorganisms and their vital activity, in general, is the effect on the production of extracellular enzymes by bacteria. For example, the suppression of QS signals may decrease the production of intracellular enzymes, which may have significant consequences for microbiological processes in nature involving extracellular enzymes of microorganisms (such as processing organic matter in the soil et cetera). Therefore, QQ processes can affect microbial communities and the whole environment [44].

Although QQ is less studied, its influence has already been considered, for example, in studies of sewage treatment in plant communities. Furthermore, it was shown that the QQ effect is less variable during the operation of the treatment facilities, while the QS effect gradually decreased. This suggests the possibility of a greatly underestimated long-term role of QQ, which may have a permanent impact on microbial communities [93].

Thus, the occurrence of QQ is determined by the continuous evolution of microorganisms. The need to exchange information in complex communities arose with the advent of the communities themselves. Coordination of activities was needed, and the language of chemical formulas was quite suitable for bacterial “communication.” As a need to clear the microenvironment of unnecessary signals, QQ is likely to have appeared along with the emergence of QS. The evolution of the QS and QQ systems goes on in parallel, and new types of informational chemical signals continue to arise during this process. In summation, we can trace the importance and breadth of the influence of QQ mechanisms that have recently begun to be actively studied. The environmental impact of the QQ effect is mediated by the importance of QS, which it directly affects, and the evolutionary context of QQ has a long history throughout the evolution of microorganisms.

## 5. Prospects for Use: The Fight against Pathogens, the Improvement of Probiotics’ Efficiency, and Plant Protection

The potential application of microorganisms capable of effectively controlling the QS of pathogens is straightforward. Many studies are aimed at finding effective QS antagonist strains for practical applications in medicine, veterinary medicine, and agriculture. There are two major areas of application of Gram-positive microorganisms to combat biofilm formation.

### 5.1. The Use of Microorganisms and Substances with QQ Activity to Protect Animals

Since QS leads to the expression of many virulence factors of pathogenic bacteria, the idea of inhibiting QS to treat bacterial infections has been in the air since the discovery of QQ. Some successes have been achieved in this field using compounds with QQ-activity, especially as an integral part of complex antibiotic therapy [21,94].

There have also been several successful trials of drugs based on AiiA, the bacillary lactonase. The whole area of QQ studies began with AiiA. In 2012, Yanan Cao et al. [95] applied bacillary lactonase AiiA by adding it to zebrafish feed, and this resulted in a significant reduction in *Aeromonas hydrophila* infection. Also, the combination of AiiA with QSI molecules made it possible to completely block the LasR/I and RhlR/I QS pathways in *Pseudomonas aeruginosa* [96]. Bacillary QQ enzymes will be discussed in more detail in the subsection on the use of Gram-positive probiotic bacteria with QQ properties.

The unexpected success of the use of QQ in the context of health care was its use to prevent overgrowth on water filters since the formation of biofilms on their surface reduces their functionality, can cause their occlusion, and lead to deterioration in the quality of pot le water. Numerous works show the effectiveness of the QQ strategy to prevent the clogging of water filters, which finds its application in industry and medicine, since the formation of biofilms on medical equipment is also a widespread problem [52,54,97]

However, it should be understood that the use of compounds with QQ activity as biocontrol agents is considered more advantageous compared to antibiotics in the context of resistance because QQ molecules do not directly kill bacteria and do not contribute to the selection of resistant forms.

Microorganisms that can implement QQ-strategies can be used as potential quenchers of QS-regulated functions in pathogenic bacteria, and they can be used as an environmentally friendly alternative to antibiotics and various fungicides in agriculture and aquaculture [98]. Antibiotic therapy exerts selective pressure on pathogens leading to the survival of resistant forms. This process is widespread due to the frequent use or overuse of antibiotics in husbandry [99] and has been shown to be accelerated in polluted soils and sediments [100].

A specific feature of the QQ strategy is that it weakens the production of virulence factors and, consequently, contributes to the destruction of a biofilm, but does not destroy the pathogen cells themselves, in contrast to the same exposure to antibacterial agents [101]. Therefore, the use of QQ probiotics may be beneficial.

It should be noted, however, that there are more and more reports that the development of resistance to QS inhibitors is still possible, and we need to use them with considerable caution [102].

According to the WHO, probiotics are “live microorganisms which when administered in adequate amounts confer a health benefit to the host” [103]. Among other things, they can be used as an alternative to antibiotics, since their antagonistic action against pathogens is multifaceted and involves many mechanisms, which excludes the rapid development of resistance by microorganisms [104]. In this light, the use of probiotics with QQ activity is of great interest, as evidenced by the many works published on this topic in the last 10 years.

Since the first enzyme with QQ activity, AiiA, was found in representatives of the *Bacillus* genus, the greatest success in the practical application of QQ was achieved with bacilli and other Gram-positive microorganisms [105].

For example, it has been shown that *B. subtilis* KATMIRA1933 produces the lantibiotic subtilisin, which significantly suppresses the formation of biofilms by the Gram-positive pathogens *Gardnerella vaginalis* and *Listeria monocytogenes*. The activity against QS of the Gram-negative bacteria was assessed using the reporter strain *C. violaceum*: the level of suppression of biofilm formation was comparable to that for Gram-positive bacteria. In the same study, the authors found that subtilosin reduces the production of QS molecule AI-2 in Gram-positive pathogens; however, the exact mechanism of this effect remains to be elucidated [49].

The probiotic strain *Bacillus* sp. QSI-1 is used to control infections in farmed fish. The study of the antagonistic properties of this strain against the pathogen *A. hydrophila* YJ-1, carried out by Zhou et al. (2019) [106], showed that co-cultivation of QSI-1 with this microorganism causes a significant decrease in the production of its virulence factors. The authors associate this result with the disruption of QS pathways of *A. hydrophila* [89,106,107].

It is also reported that *Bacillus licheniformis* T-1 exhibits probiotic and QQ properties both in vitro and in vivo. Next-generation sequencing data suggest that this strain contains the *ytn*P gene encoding acyl-homoserine lactone metallo-β-lactamase, a potential quorum-quencher. QQ activity is confirmed in studies with the reporter strain *C. violaceum* ATCC12472. In addition, with intraperitoneal administration of T-1 to zebrafish infected with *A. hydrophila* cb15, the pathogenicity of this microorganism was significantly reduced, and the relative survival rate of fish reached 70% [108].

Ghanei-Motlagh et al. (2019) [109] isolated 10 bacterial species from the intestines of barramundi capable of degrading both short-chain and long-chain AHLs associated with common fish pathogens—*V. harveyi* and *V. alginolyticus*. Moreover, the researchers tested the probiotic potential of the isolated species in vitro and concluded that at least 2 of the tested strains (*B. thuringiensis* QQ1 and *B. cereus* QQ2) meet the criteria for probiotic bacilli such as spore formation, exoenzymes secretion, low pH tolerance, the ability to adhere to the mucosa, and safety for fish. The same authors determined that *B. thuringiensis* QQ1 and *B. cereus* QQ2 can significantly reduce the production of *V. alginolyticus* virulence factors (amylase, gelatinase, and protease), as well as inhibit its ability to form biofilms. Also, both probiotics increased Asian seabass survival during *V. alginolyticus* infection [110]. 

In addition to the spore-forming probiotics discussed above, some progress has been made in the use of probiotic lactobacilli. *Lactobacillus brevis* 3M004 (reclassified as *Levilactobacillus brevis*) is capable of cleaving acyl-homoserine lactones, but its therapeutic potential needs further verification [111]. *L. plantarum* PA100 is able to inhibit the production of AHL, elastase, and biofilm formation in *P. aeruginosa*, which is resistant to several antibiotics [112]. Strains *L. casei* ATCC 393, *L. reuteri* ATCC 23272, *L. plantarum* ATCC 14917, and *L. salivarius* ATCC 11741 demonstrated an inhibitory effect on biofilm formation and gene expression of the QS system (vicR and comD) in *Streptococcus mutans*, however, the exact mechanisms of this effect are still unknown [113]. *Lactobacillus reuteri* RC-14, a vaginal isolate, when co-cultured with *Staphylococcus aureus* MN8, inhibited transcription from the promoters of the agr QS genes (Ptst, P2, and P3). This led to the repression of the synthesis of virulence factors, including exotoxin toxic shock syndrome toxin-1 (TSST-1). This effect on expression was associated with the signal peptides cyclo (L-Phe-L-Pro) and cyclo (L-Tyr-L-Pro) [55].

In addition to all of the above, there are sporadic reports on the use of probiotic enterococci. For example, *Enterococcus faecium* QQ12 has shown effective degradation of N-AHL produced by the pathogen *Aeromonas hydrophila*, demonstrating the potential of *E. faecium* as a QQ strain [114].

Thus, we see that the potential for developing probiotics with QQ activity is truly enormous, and the sources of new probiotic strains for further selection and use can vary widely.

### 5.2. Symbiotic and Mutualistic Microorganisms Capable of Protecting Plants from Pathogens, Exhibiting QQ Properties

Plants and bacteria have a long history of coevolution, and their ecological relationships range from symbiosis and even mutualism to parasitism. The situation is especially difficult in the rhizosphere, where the networks of interactions are truly immense. In this regard, the issue of using microorganisms to combat plant parasites is quite acute. Since many plant pathogens are capable of forming biofilms, and the use of compounds active against a wide range of microorganisms can damage beneficial bacteria in the rhizosphere, the use of drugs with QQ activity seems to be a logical solution [115].

Dong et al. (2000) [116] were the first to try to use QQ for plant protection. They modified the plant pathogen *P. carotovorum* with the *aiiA* lactonase gene isolated from *Bacillus* sp. 240B1. As a result of this intervention, the secretion of QS autoinducers and other virulence factors was significantly reduced, and the pathogenicity on potato, eggplant, Chinese cabbage, carrot, celery, cauliflower, and tobacco was weakened. In 2001, the same authors transformed tobacco and potatoes with the *aiiA* gene. The transformed plants showed significant resistance to *P. carotovorum* [94]. Similar studies with *aiiA* and other lactonase genes were subsequently carried out by other authors [117,118].

Gram-positive microorganisms are also used directly to combat phytopathogens through QQ. Yi-Hu Dong et al. (2004) [119] tested *B. thuringiensis* for the prevention of potato soft rot caused by *P. carotovorum*. It was shown that bacilli do not directly affect the growth of the bacteria, but inhibit the synthesis and accumulation of AHL, which leads to a substantial decrease in the number of infection cases. Also, *Bacillus cereus* Si-Ps1, an endophyte isolated from the leaves of *Citrus sinensis* and *C. sinensis* var. Thomson’s, according to a study by Akbari Kiarood et al. (2020) [110], produces an analog of lactonase *aiiA*, thereby inhibiting biofilm formation in the bacterium *Pseudomonas syringae* pv. syringae (Pss) B728a. Since Si-Ps1 is an endophyte, its use for plant protection presents no complications.

Proof of the prospects of QQ for plant protection is seen in modern screening studies, the authors of which are looking for strains with QQ-activity in the rhizosphere of different plants. For example, Fatemeh Alinejad et al. (2020) [120] isolated several soil microorganisms, including *Bacillus pumilus*, capable of degrading acyl-homoserine lactone while reducing soft rot in potato tubers by 98% compared to controls.

However, it should be understood that intervention into the complex environmental relations in the rhizosphere often comes at a cost, and sometimes we may not precisely get the effect we expect since QS systems are not only used by pathogens. Noteworthy in this context is the *Bacillus cereus* strain U92, which was isolated from the rhizosphere of tomato. It has shown both in vitro high efficiency in degradation of a broad spectrum of AHL and in vivo reduction in the incidence of soft rot on potato tubers and crown gall on tomato roots. On the other hand, this led to a 75% drop in QS-dependent pyocyanin synthesis by *P. aeruginosa*, a plant growth-promoting bacterium [121]. This again emphasizes that any intervention in complex microbial communities, even more so in cross-kingdom interactions, must be carried out with great care.

## 6. Conclusions

Quorum-quenching activity has been forged by evolution as an effective mechanism of interspecies antagonism. Many pathways underlying pathogenicity and resistance to antimicrobial agents are QS-mediated, thus, the use of QQ is a promising way to fight against pathogenic bacteria. Further studies can lead to the development of new pharmaceuticals with a targeted mode of action.

The main mechanisms that Gram-positive bacteria use to inhibit QS are inhibition or degradation of autoinducers. Representatives of the *Bacillus* genus use AHL lactonases, AHL acylases, AHL oxidases, and reductases to degrade AHL; lactic acid bacteria also degrade signaling molecules but are also able to influence the expression of genes involved in QS. Actinobacteria produce lactonases, AHL acylases, oxidoreductases, and also cyclic lipopeptide acylases, which allow them to disrupt lipopeptide signaling.

The QQ-based interactions between different groups of bacteria can also provide new insights into the mechanisms of probiotic activity since most probiotics are gram-positive bacteria.

The studies of QQ in Gram-positive bacteria can improve our understanding of microbial ecosystems functioning in all types of environments—from soils and sediments to the intestinal microbiome. The impairing of QS signals by Gram-positive bacteria can help them to compete for these microenvironments with more rapidly growing Gram-negative species. Its ecological significance can be put in line with complex pathways of secondary metabolites and antibiotic production in Actinobacteria. The growing body of evidence demonstrating the existence of QQ in lactobacilli and bifidobacteria can lead to an assumption that the prevalence of these groups of microorganisms in the intestine is at least partly due to the QQ-mediated competition with Gram-negative species. Further studies are needed to evaluate the role of QQ in the regulation of bacterial communities in various environments.

## Figures and Tables

**Table 1:** AHL enzyme categories and their molecular mechanism.

Enzyme Categories	Mechanism of AHL Degradation
AHL lactonases	Hydrolysis of the AHL lactone ring to form the corresponding N-acyl homoserine. This process can also occur spontaneously in the presence of an alkaline pH and can be reversed when the pH is acidified
AHL acylases	Degradation of the AHL amide bond and generation of the corresponding free fatty acid and a lactone ring
AHL oxidoreductases	Modification (oxidation) and subsequent degradation of signal QS molecules

**Table 2:** Strains of lactic acid bacteria—QS antagonists.

Strain	Mechanism	Against	References
*L. acidophilus* 30SC	AI-2 Inhibition	*E. coli* O157:H7	[51]
*L. plantarum* SBR04MA	N-Hexanoyl-L-homoserine lactone (6-HSL) inhibition	Microbiota of activated sludge	[54]
*L. plantarum* CY-1	HSL degradation (without details).	*A. sobria*	[67]
*C. crustorum* ZHG 2-1	Dose-dependent degradation C4- and 3-oxo-C12- HSL	*P. aeruginosa*	[58]
Cell-free acidic supernatants *L. lactis* NCDC 309, *L. rhamnosus* MTCC 5897, *L. rhamnosus* MTCC 5857, *L. fermentum* MTCC 5898, *L. acidophilus* NCDC 15, *L. delbrueckii subsp. lactis*, *L. plantarum* NCDC 372,	Inhibition of biofilm formation, elastase, and expression of *lasI* and *rhlI*	*P. aeruginosa*	[59]
Cell-free neutral supernatants of some of the strains described above	Depending on the strain, both the lack of effect and the stimulation of some QS processes (an increase in the expression level of QS-related genes, elastase activity) were observed	*P. aeruginosa*	[59]
*L. reuteri* RC-14	Inhibition of *agr* gene expression by cyclic dipeptides cyclo(L-Phe-L-Pro) and cyclo(L-Tyr-L-Pro)	*S. aureus*	[55]
*L. plantarum*, *P. acidilactici*	Reduce expression of genes *cidA*, *icaA*, *dltB*, *agrA*, sortaseA, and *sarA* involved in biofilm formation	*S. aureus*	[65]
*L. plantarum* M.2, *L. curvatus* B.67	Inhibition of *agr* genes	*Listeria monocytogenes*	[65]
Heat-treated supernatant *L. fermentum* Lim2	Inhibition of *agr* genes	*C. difficile*	[66]

**Table 3:** Strains of Actinobacteria—QS antagonists.

Strain	Mechanism	Against	References
*Rhodococcus pyridinivorans* AI4	3-oxo-C6- N-acyl homoserine lactone degradation	*Pectobacterium carotovorum*	[71]
*Glutamicibacter nicotianae* AI5a	3-oxo-C6- N-acyl homoserine lactone degradation	*Pectobacterium carotovorum*	[71]
*Arthrobacter* sp. IBN110	3-oxo-C6- N-acyl homoserine lactone degradation	*Pectobacterium carotovorum* N98	[79]
*Microbacterium testaceum* StLB037	C10-HSL degradation	*Pectobacterium carotovorum*	[76]
*Micromonospora* sp. TAV14	Disruption of rhl-regulated QS	*P. aeruginosa* PAO1	[77]
*Streptomyces* sp. NIO 10068, *Streptomyces* sp. NIO 10058	Disruption of rhl-regulated QS	*P. aeruginosa* ATCC 27853	[80]
*Rhodococcus erythropolis* R138	3 types of AHL degradation enzymes, AHL lactonase, acylase, and oxidoreductase	*Pectobacterium atrosepticum* CFBP 6276	[81,82]

## Data Availability

Not applicable.
